# Evaluation of urinary hydroxyproline and creatinine level in patients with benign mandibular odontogenic tumor

**DOI:** 10.1002/cre2.392

**Published:** 2021-01-25

**Authors:** Chidozie I. Onwuka, Chibuzo C. Uguru, Chidinma I. Onwuka, Ambrose E. Obiechina

**Affiliations:** ^1^ Department of Oral and Maxillofacial Surgery University of Nigeria Teaching Hospital Enugu Nigeria; ^2^ Department of Obstetrics and Gynaecology University of Nigeria Teaching Hospital Enugu Nigeria; ^3^ Department of Oral and Maxillofacial Surgery University of Port‐Harcourt Teaching Hospital River State Port Harcourt Nigeria

**Keywords:** colorimetric analysis, creatinine, odontogenic tumors, urinary hydroxylproline

## Abstract

**Background:**

Odontogenic tumors are relatively common oro‐facial tumors seen in our environment with challenges encountered with management in terms of inadequate infrastructure and high cost of treatment. They are often associated with bone resorption with concomitant collagen degradation and excretion of their by‐products in serum or urine. The aim of this present study was to evaluate urinary hydroxyproline level in patients with benign mandibular odontogenic tumors.

**Materials and methods:**

Twenty‐two consecutive patients with histologically diagnosed mandibular odontogenic tumors were recruited. Twenty‐two controls who matched the study group for sex and age were also recruited. The study group had CT‐Scan of their lesions done. All participants were required to fast 12 hours overnight and their early morning second void urine collected between 7 a.m and 8 a.m. The collected urine samples were stored frozen at −20°C until analysis. Colorimetric method of analysis of urinary hydroxyproline and creatinine were done using Biovision hydroxyproline kit and Randox creatinine kit, respectively. The results were recorded as urinary hydroxyproline alone (μg/μl) and as urinary hydroxyproline/creatinine ratio.

**Results:**

The mean age of the participants was 28.45 ± 6.8 years. The mean duration of the tumors in the study group was 5.9 ± 4.4 years. A mean urinary hydroxyproline/ creatinine ratio of 0.081 ± 0.129 was noted in the study group as compared to 0.016 ± 0.006 that was noted among healthy Nigerian who served as controls in the study.

**Conclusion:**

There was a significant increase in urinary hydroxyproline level in patients with odontogenic tumors when compared with healthy Nigerians.

## 1 INTRODUCTION

Odontogenic tumors are relatively common oro‐facial tumors seen in our environment (Akinmoladun et al., 2010; Aregbesola et al., 2018).These tumors are associated with bone resorption, osteosclerosis, cortical bone swelling, destruction, and calcification which can be seen on radiological imaging (Qian & Huang, 2010; Yamada et al., 2016). Disease induced bone resorption is associated with increase in biochemical marker levels which reflect the rate of bone metabolism (Lipton et al., 1998). However, changes in biochemical markers of bone metabolism are not disease specific rather they reflect changes in bone metabolism (Seibel, 2005). Their measurements are usually associated with variable techniques and application which typically are non‐invasive and can be conveniently measured in urine and blood (Rissanen, 2013). Biochemical markers of bone resorption include hydroxyproline which is an amino acid derivative from the post‐translational hydroxylation of proline that is released into the circulation during bone resorption from collagen degradation (Hlaing & Compston, 2014). Measurement of urinary level or concentration of hydroxylproline is considered as an accurate measure of bone resorption activity even though some limitations of its use have been reported (Gotfredsen et al., 2001; Indumati & Patil, 2010; Watts, 1999) These limitations include non‐specificity for bony lesions and the fact that their levels are subject to dietary variables (Gotfredsen et al., 2001; Hlaing & Compston, 2014). However, overnight fast or 24 hours fast from gelatine containing foods eliminates dietary aspect of variability in its measurement (Szulc & Delmas, 2008). Analysis of urinary hydroxyproline is usually performed either by colorimetric method or high performance liquid chromatography (HPLC) (Simsek et al., 2004). Urinary measurement of hydroxyproline is non‐invasive and can be performed at frequent intervals with less risk to patients as compared to plain radiographs and CT‐scans. It is also an alternative way of assessing bone cellular activity and is the most performed measure of bone resorption activity with the longest history of use (Carvo et al., 1996). Studies in literature have evaluated the role of urinary hydroxyproline in different bone disease conditions (Ali et al., 2017; Manohari et al., 2015).

In spite of the fact that bone metabolism biomarkers measurements are cheap and noninvasive means of monitoring bone diseases, its application is not widely explored in scarce resources economy like Nigeria. Odontogenic jaw tumors are prevalent among Nigerians and there has never being any study evaluating the usefulness of bone resorption markers in the management of these patients. This study was therefore aimed at evaluating the clinical usefulness of these markers of bone metabolism such as hydroxproline in the management of patients with odontogenic tumors in Nigerians with the view of finding cheaper and less risky alternative to frequent bone imaging.

## 2 MATERIALS AND METHODS

This was a prospective cross sectional analytical study conducted at University of Nigeria Teaching Hospital involving a total of 44 participants between the ages of 18 years and 40 years. Participants were divided into two groups: Group A (study group) consisted of 22 consenting participants with mandibular odontogenic tumors and group B (control group) consisted of 22 healthy participants who matched the study group in sex and age. Participants used in the study were comprehensively assessed; bio‐data and medical history were taken and physical examination done for each participant as well as CT‐scan imaging for the study group. Data obtained were entered into a proforma designed for the study. Patients who presented with other known bone destructive lesions (inflammatory or metabolic) or had fractures of any bone within the last 1 year as well as other conditions such lactating mothers, postmenopausal patients, patients below 18 years and above 40 years and patients on steroid therapy/ oral contraceptives were excluded from the study. Pathological result of incisional biopsy of the lesions was documented for each participant in the study group. Participants were admitted into the ward to ensure that they fasted overnight for at least 12 hours before sample collection. Early morning second urine voided between 7 a.m and 8 a.m. were collected in a plastic urine bottle without preservative. This provides a reliable data on bone degradation (Smith et al., 2004).The samples were well labeled and stored frozen in the laboratory in a freezer with thermometer maintained at ‐20°C. The collected stored urine samples were thawed and analyzed for urinary hydroxylproline and urinary creatinine. Hydroxyproline research kit (Biovision Inc. 155S Milpitas Blvd, Milpitas, CA, 95035 USA) was used in the colorimetric analysis of urinary hydroxyproline. Frozen urine samples were first thawed and stirred. Hundred microliters of each calibrator, control and specimen samples were pipetted into corresponding labelled wells in precise tight Teflon capped vial. Hundred microliters of concentrated hydrochloric acid (~12 N) was added to each capped vial and hydrolysis was done at 120°C for 3 hours. They were incubated at room temperature for 5 minutes. Hundred microliters of DMAB reagent was then added to each well and incubated for 90 minutes at 60°C. The plates were read on a micro plate reader at 560 nm. The reading from the standard solution was used to generate linear graph from which sample readings were gotten.

The thawed urine samples were also analyzed for urinary creatinine using modified Jaffe's method. Creatinine analysis was done using Creatinine research kit (Randox Laboratories Limited, 55 Diamond Road Crumlin, County Antrim BTY29 4QY United Kingdom). The working reagent was prepared by mixing equal volumes of reagent 1a (Picric acid) and reagent 1b (Sodium hydroxide). Urine samples were prepared by making 1:50 dilution. Two milliliters of working reagent was added to 0.2 ml standard (Std) solution and mixed, 30 seconds after the mixture the absorbance (A1) reading was taken then, exactly 120 seconds after initial mixture absorbance (A2) reading was taken. This generated a standard reading from which the sample reading will be calculated from. The creatinine of each sample was calculated using the formula: A2 — A1/ Std2 — Std1 × Std concentration × 50.

Volume of bone destruction was calculated mathematically using the formula V = LS X AP X TS X 0.52 (cm) ^3^. Where V is the volume of bone destruction, LS is the longitudinal section of the lesion, AP is the antero‐posterior section, TS is the transverse section of the lesion, and 0.52 is the constant value. The hounsfield unit of the mass was also gotten to know the density of the lesion.

### 2.1 Statistical analysis

The data collated was analyzed using IBM Statistical Package for the Social Sciences (SPSS) Statistics for windows version 21 (IBM Corp., Armonk, N.Y., USA). Continuous variables were summarized using means and standard deviations while categorical variables were summarized using frequency and percentages. Means of continuous variables were compared using student's t test and ANOVA. Relationship between continuous variables was done using Linear Regression and Pearson correlation analysis. All tests were significant at probability level (*p*) of <0.05.

## 3 RESULTS

Forty‐four participants were recruited in the study and 44 urine samples were analyzed for urinary hydroxyproline levels and urinary creatinine levels. The results were recorded as urinary hydroxyproline levels alone and urinary hydroxyproline/creatinine ratio.

**FIGURE 1:**
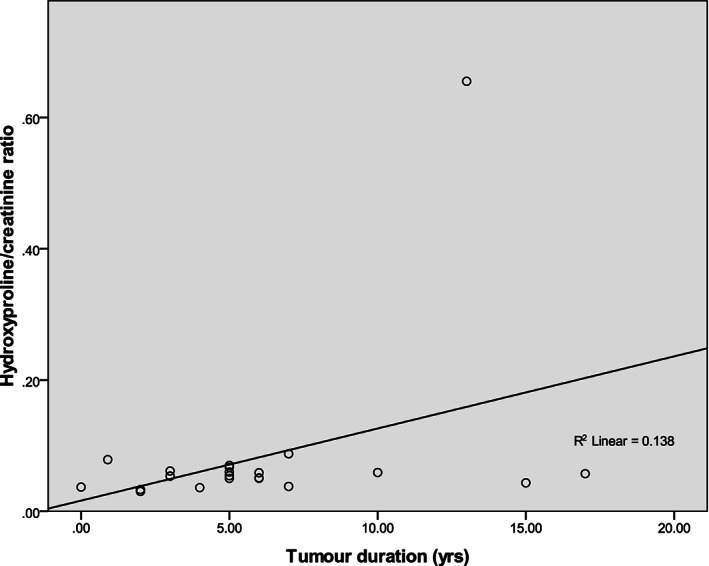
A scatter plot of relationship between the level of urinary hydroxyproline/ creatinine ratio and tumor duration

Sociodemographics of the study showed that the gender distribution of the participants was 15 males (68.2%) and 7 females (31.8%), respectively for each group. The mean ages of participants were 28.45 ± 6.8 years for each group. Age group of 20–24 years accounted for 50.0% of subjects while the age group of 25–29 years and 30–34 years accounted for 4.5% and 13.6%, respectively. Participants within the age group of 35–39 years and > 39 years accounted for 13.7% and 18.2%, respectively (Table 1). The mean duration of the tumors in the study participants was 5.90 ± 4.4 years with the minimum tumor duration of 2 years and maximum tumor duration of 17 years. The mean volume of bone destruction in the study group was 446.682 ± 7.18 cm^3^. Ameloblastoma accounted for 86.5% of the lesions seen with calcifying epithelial odontogenic tumor, Odontogenic keratocyst (formally KCOT) and odontogenic fibroma accounting for 4.5%, respectively (Table 2). A mean value of 0.020 ± 0.013 μg/μl for urinary hydroxyproline and 0.016 ± 0.006 for urinary hydroxyproline/creatinine ratio was noted for the control group. The mean urinary hydroxyproline value of 0.100 ± 0.059 μg/μl of study group was significantly different from the mean urinary hydroxyproline value of 0.020 ± 0.013 μg/μl of the control group (*p* < 0.001). The mean urinary hydroxyproline/creatinine ratio value of 0.081 ± 0.129 for the study group was significantly different (*p* < 0.05) from that of the control group with a value of 0.016 ± 0.006 with *p* = 0.023 (Table 3). There were no significant differences between males and females among the control group (*p* = 0.334) for urinary hydroxyproline levels and for urinary hydroxyproline/creatinine ratio, *p* = 0.604. There were also no significant differences between males and females of the study group, *p* = 0.381 for urinary hydroxyproline levels and p = 0.378 for urinary hydroxyproline/creatinine ratio (Table 4). There was no significant difference between the age groups in values of urinary hydroxyproline and urinary hydroxyproline/creatinine ratio values in both study and control groups (Tables 5 and 6). Also no significant difference was noted in the volume of bone destruction between subjects with follicular and plexiform variants of ameloblastoma (Table 7) and no significant relationship between urinary hydroxyproline level, urinary hydroxyproline/creatinine ratio and the volume of bone destruction in the study group (Table 8). There was no significant relationship between the hydroxyproline/creatinine ratio and tumor duration with the scattered plot showing cluster of points that exhibits no regular pattern (Figure 1).

**TABLE 1:** Socio‐demographic characteristics of subjects

	Subject *n* (%)	Control *n* (%)
**Sex**		
Male	15 (68.2)	15 (68.2)
Female	7 (31.8)	7 (31.8)
**Age group (years)**		
20–24	11 (50.0)	11 (50.0)
25–29	1 (4.5)	1 (4.5)
30–34	3(13.6)	3 (13.6)
35–39	3 (13.7)	3 (13.7)
>39	4 (18.2)	4 (18.2)
**Educational status**		
Primary	8 (36.4)	0 (0.0)
Secondary	11 (50.0)	3 (13.6)
Tertiary	3 (13.6)	19 (86.4)
**Occupation**		
Trader	5 (22.7)	0 (0.0)
Student	9 (40.9)	10 (45.5)
Artisan	2 (9.1)	0 (0.0)
Unemployed	4 (18.2)	0 (0.0)

**TABLE 2:** Study group biopsy and ameloblastoma variants

	*N* (%)
Odontogenic fibroma	1 (4.5)
Ameloblastoma	19 (86.5)
Odontogenic Keratocyst (KCOT)^a^	1 (4.5)
Calcifying epithelial odontogenic tumor	1 (4.5)
**Ameloblastoma variants**	
Follicular	19 (89.0)
Plexiform	2(11.0)

^a^ KCOT, Keratocystic odontogenic tumor formally the name for odontogenic keratocyst as at the time of the study.

**TABLE 3:** Mean urinary hydroxyproline comparison between the study group and control group

	Study Mean ± SD	Control Mean ± SD	t	*p* value
Hydroxyproline (μg/μl)	0.100 ± 0.059	0.020 ± 0.013	6.108	<0.001
Creatinine (μg/μl)	0.76 ± 0.98	1.25 ± 0.68	1.973	0.055
Hydroxyproline/creatinine	0.081 ± 0.129	0.016 ± 0.006	2.355	0.023

**TABLE 4:** Mean urinary hydroxyproline comparison between male and female of the study group and control group

	Male Mean ± SD	Female Mean ± SD	t	*p* value
**Study group**				
Hydroxyproline (μg/μl)	0.092 ± 0.059	0.117 ± 0.062	0.896	0.381
Hydroxyproline/creatinine ratio	0.098 ± 0.155	0.045 ± 0.011	0.901	0.378
**Control group**				
Hydroxyproline (μg/μl)	0.019 ± 0.012	0.025 ± 0.017	0.990	0.334
Hydroxyproline/creatinine ratio	0.016 ± 0.006	0.017 ± 0.003	0.528	0.604

**TABLE 5:** Mean urinary hydroxyproline comparison across the age groups of the male and female control group

	Age (years)						
	20–24 Mean ± SD	25–29 Mean ± SD	30–34 Mean ± SD	35–39 Mean ± SD	>39 Mean ± SD	F	*p* value
**Male**							
Hydroxyproline (μg/μl)	0.01 ± 0.01	0.02 ± 0.02	0.01 ± 0.01	0.02 ± 0.01	0.02 ± 0.01	0.946	0.474
Hydroxyproline/creatinine	0.02 ± 0.01	0.01 ± 0.01	0.01 ± 0.01	0.02 ± 0.03	0.02 ± 0.01	0.613	0.662
**Female**							
Hydroxyproline (μg/μl)	0.02 ± 0.02	0.03 ± 0.02	‐	0.02	‐	0.048	0.954
Hydroxyproline/Creatinine	0.02 ± 0.01	0.02 ± 0.01	‐	0.02	‐	0.986	0.469

**TABLE 6:** Mean urinary hydroxyproline comparison across the age groups of the male and female of the study group

	Age (years)						
	20–24 Mean ± SD	25–29 Mean ± SD	30–34 Mean ± SD	35–39 Mean ± SD	>39 Mean ± SD	F	*p* value
**Male**							
Hydroxyproline (μg/μl)	0.12 ± 0.07	0.03	0.06 ± 0.017	0.09 ± 0.09	0.09 ± 0.09	0.666	0.630
Hydroxyproline/creatinine	0.06 ± 0.01	0.08	0.05 ± 0.01	0.07 ± 0.03	0.25 ± 0.35	0.892	0.503
**Female**							
Hydroxyproline (μg/μl)	0.12 ± 0.07	‐	‐	0.16	0.06	0.575	0.603
Hydroxyproline/Creatinine	0.05 ± 0.01	‐	‐	0.05	0.03	0.887	0.480

**TABLE 7:** Mean comparison of volume of bone destruction between subjects with follicular and plexiform variants of ameloblastoma

	Follicular	Plexiform		
	Mean ± SD	Mean ± SD	T	*p* value
Volume of bone destruction (cm^3^)	666.48 ± 6.03	1095.70 ± 1.36	0.855	0.404

**TABLE 8:** Relationship between urinary hydroxyproline level and volume of bone destruction

	Correlation coefficient (R)	Coefficient of determination (R^2^)	Regression coefficient (B)	*p* value
Hydroxyproline (μg/μl)	0.381	0.145	0.000	0.080
Hydroxyproline/creatinine ratio	0.056	0.003	‐ 0.000	0.805

## 4 DISCUSSION

Challenges of providing adequate healthcare services in resource scarce economy have necessitated the need to explore cheaper and easier way of monitoring patients with bone diseases where possible. Measurement of biochemical markers of bone metabolism such as urinary hydroxyproline offers a cheaper and less invasive means of assessing bone cellular activity at more frequents intervals as compared to other means of bone assessments (Ugar & Karaca, 1999) with fasting urinary hydroxyproline/creatinine ratio adjudged to be a better indicator of bone resorption than urinary hydroxyproline alone (Sarvari et al., 2013).

In our study, the mean urinary hydroxyproline/creatinine ratio level of 0.016 ± 0.006 was noted among healthy Nigerians who served as control and this was similar to values reported for healthy individuals in the literature (George, 2003; Sarvari et al., 2013). Accordingly, we noted a significant difference in urinary hydroxyproline value and urinary hydroxyproline/ creatinine ratio between the study group and the controls. This difference in the values of urinary hydroxyproline and urinary hydroxyproline/creatinine ratio between the study group and the controls could be attributed to the osteolytic changes as seen on the CT‐scan of the study group participants noting no other lesion(s) other than a mandibular tumor among the study group during comprehensive medical assessment. These osteolytic changes are due to bone destruction which leads to collagen degradation and subsequent liberation of hydroxyproline which is not utilized again in collagen synthesis but excreted in urine (Mukhopadhyay et al., 2011). This process may have accounted for the difference in urinary hydroxyproline level between the study and control groups in our present study. This difference may be explored in conservative management of odontogenic tumors where frequent measurements of urinary hdyroxylproline may be used to monitor bone resorption activity thus serving as a less risky and cheaper alternative to serial ct scans and other expensive bone imaging in monitoring tumor reoccurrence. However, measurement of urinary hydroxyproline cannot replace completely the use of bone imaging in management of odontogenic but may predict when to use them following less aggressive management of odontogenic tumors since changes in their levels are noted long before radiographic manifestation of the bone disease. The use of the difference in baseline urinary hydroxylproline levels between study group and control as well as before and after treatments have been employed in monitoring of metastasis to bone from soft tissue tumors, metabolic bone diseases and bone fracture union (Ali et al., 2017; Manohari et al., 2015; Sarvari et al., 2013).

There was no significant gender difference in urinary hydroxyproline/creatinine ratio noted in this study which is similar to reports in literature (George, 2003; Paroni et al., 1992). There was also no significant difference in urinary hydroxyproline/ creatinine ratio across the age groups of the study group and controls in this study. This could be attributed to the fact that there is no significant age related bone loss in males as compared to females (Sarvari et al., 2013). In females, the present study observed no significant difference among the age groups; this could be to the fact that the females in the study were premenopausal. Many studies noted an increase in urinary hydroxyproline/creatinine ratio among females as they age from premenopausal to peri and postmenopausal age (Indumati et al., 2007; Sachdeva et al., 2005). They attributed this increase in urinary hydroxyproline /creatinine ratio to bone loss associated with menopause.

Further findings in our study noted ameloblastoma as the commonest odontogenic tumor seen which is similar to findings in other Nigerian studies (Aregbesola et al., 2018; Ladeinde et al., 2005). There was significant bone resorption associated with the lesions seen in this study even though there was no significant difference in bone destruction between the two variants of ameloblastoma seen in our study.

In conclusion, our study showed that the urinary hydroxyproline levels in study population of healthy Nigerians were within the normal values as reported in previous studies in Nigeria and from other countries. Significant increase in urinary hydroxyproline level was noted in patients with benign odontogenic tumors when compared with healthy Nigerians and this suggests that its measurement may play a useful role in management of patients with odontogenic tumors. Small sample size and analytical variability associated with hydroxproline measurements are limitations that may have affected our study. However, further multicentre studies involving other biomarkers of bone resorption are recommended.

## FUNDING

None.

## CONFLICT OF INTEREST

The author declares that there is no conflict of interest that could be perceived as prejudicing the impartiality of the research reported.

## ETHICS STATEMENT

This study was approved by the Ethics committee of the University of Nigeria Teaching Hospital, Ituku‐Ozalla, Enugu.

## PATIENT CONSENT

Written Informed consent forms were signed by all patients who agreed to participate in the study.

## AUTHOR CONTRIBUTION

All the authors contributed to the design and conceptualization of the study and writing of the manuscript.

## Data Availability

The data that support the findings of this study are available from the corresponding author upon reasonable request.
